# A decade of an HIV workplace programme in armed conflict zones; a social responsibility response of the International Committee of the Red Cross

**DOI:** 10.1186/s12995-016-0119-4

**Published:** 2016-05-31

**Authors:** Stéphane Du Mortier, Silas Mukangu, Charles Sagna, Laurent Nyffenegger, Sigiriya Aebischer Perone

**Affiliations:** International Committee of the Red Cross, Geneva, Switzerland

**Keywords:** HIV/AIDS, Social responsibility, ICRC, Workplace programme, Conflict, Humanitarian, Fragile States

## Abstract

The International Committee of the Red Cross (ICRC) works in fragile States and in armed conflict zones. Some of them are affected by the HIV pandemic. Within the framework of its social responsibility programme concerning HIV affecting its staff members, the organization has implemented an HIV workplace programme since 2004.

We carried out a retrospective analysis over 10 years. Data collected were initially essentially qualitative and process-oriented, but were complemented over the years by data on annual voluntary counselling and testing (VCT) uptake and on direct annual costs covering awareness, testing and antiretroviral therapy.

The number of people covered by the programme grew from none in 2003 to 4,438 in 2015, with an increase in annual VCT uptake over the years increasing from 376 persons (14 %) in 2007 to 2,663 in 2015 (60 %).

Over the years, the services were expanded from awareness raising to bringing VCT to the workplace, as well as offering testing and health coverage of other conditions and innovative approaches to facing challenges linked to situations of violence.

Within its social responsibility framework, the ICRC has shown the importance and feasibility of a workplace HIV programme in conflict zones. A sustainable workplace programme in these conflict settings requires constant adaptation, with regular follow-up given the relatively high turnover of staff, and ensuring sustainable stocks of condoms and antiretroviral drugs.

## Background

The use of war as a continuation of diplomacy when pursuing interest is becoming more acceptable, as systems of states are changing. State driven, inter-communal and urban violence diffuses into society destroying social fabrics. One of the definition of fragile states is weak capacity to carry out basic state functions and weak institutions. Humanitarian actors need to be agile and provide access and basic health services in places with health system failure.

The International Committee of the Red Cross (ICRC) is an independent, neutral organization ensuring humanitarian protection and assistance to victims of armed conflict and other situations of violence. It acts in response to emergencies and at the same time promotes respect for international humanitarian law and its integration into national law. Based in Geneva, Switzerland, it has a workforce of about 12,000 people in 80 countries, of whom many are affected by the HIV pandemic.

HIV workplace programmes (WPP) have been launched as new approaches in the fight against HIV [1], since HIV primarily affects populations of working age [2] and 54 % of people living with HIV (PLHIV) worldwide do not know that they are infected [3].

Publications on HIV at the workplace began to appear from 2004, many from southern Africa [4, 5], which illustrated some specific characteristics of the epidemic including higher prevalence of HIV among short-contract workers compared to permanent employees and among people in occupations with low wages. Prevalence peaks between 30 and 39 years for men and at younger ages for women [6].

In June 2013, the International Labour Organization (ILO) launched its “VCT@work initiative”. Michel Sidibé, UNAIDS Executive Director, also highlighted the importance of the fight to reach the target of 5 million workers tested at work by 2015: “*We need to tell them that HIV is no longer a death sentence. They must know their status. The days when they had to hide are over. HIV is a chronic, treatable disease, and treatments are becoming less toxic*” [7].

As response to HIV affecting its staff members, the ICRC has been implementing an HIV WPP since 2004. This article reports the successes, challenges and lessons learnt from a decade of running an HIV WPP in armed conflict zones and fragile States.

## ICRC and its HIV workplace programme

In 2004, 48 % of the ICRC’s operational budget was spent in Africa [8], where the highly prevalent HIV pandemic had a great impact on the ICRC’s workforce and its operational capacity. In order to promote the health of its staff and in line with its organizational social responsibility, ICRC representatives benchmarked other renowned WPPs, such as those of the German Society for International Cooperation (GIZ) and the Heineken breweries, and started a pilot HIV WPP which quickly expanded in numerous countries. The implementation of an HIV WPP, as well as its benefits for staff, also had economic consequences by reducing replacement costs, retaining know-how and sending a signal to the labour market, as it is more efficient to implement an HIV WPP than to lose and have to replace deceased staff members and to be obliged to develop the capacity of newly hired staff. The ICRC staff health unit wrote the HIV WPP policy, which was validated in 2006 by the ICRC Assembly, and which is in line with the ILO code of practice on HIV/AIDS and the relevant ILO guidelines [9–11]. This policy guarantees non-discrimination, confidentiality and no link between testing, status and employment. It also respects a recent ILO recommendation of 2010 [12] and ISO 26000, concerning guidance on social responsibility [13].

The desired impact of the ICRC HIV WPP is that there will be no new HIV infections among staff. The three pillars of the programme are a supportive environment, permanent access to quality HIV testing, and proper care and treatment.

The annual objectives are: (1) to increase annually the number of staff and family members who know their HIV status, with 100 % coverage of HIV testing for all pregnant women; and (2) to guarantee that HIV-infected staff and family members have access to quality care and treatment. We illustrate, below, the difficulties of guaranteeing permanent access to preventive services (especially testing) in situations of violence and armed conflicts.

### Leadership of the HIV WPP

Numerous articles document the fact that one of the key factors for a successful HIV WPP is leadership [2, 14–19] resulting from a synergy between motivated, responsible personnel at three levels: national health focal points (HFPs), regional coordinators and headquarters staff.

In each country, one HFP is responsible for implementation of the WPP and for ensuring access to health services. The HFP is bound by medical confidentiality in relation with her/his colleagues from the human resources (HR) department and has responsible for peer educators (PE) selected by employees. HFPs are trained to address stigma and discrimination, to promote HIV awareness, to refer staff to voluntary counselling and testing (VCT) and to guarantee permanent condom availability, post-exposure kits and management of medical waste. They report to line managers organized in an “HIV committee”, which meets at least once a year and which includes the senior management of the delegation concerned. Its members are sensitized and important boosters of the WPP especially in addressing stigma and discrimination, providing time for HIV awareness sessions and allowing VCT at the workplace, as already documented in different contexts [19, 20].

Three regional coordinators, based in Nairobi and Dakar, provide strong leadership through annual country visits and permanent technical support. At headquarters level, the health unit is available to guarantee permanent support to the programme.

### Creating a supportive environment

We regularly conducted locally adapted information and education sessions on HIV/AIDS among employees by using health professionals for question-and-answer sessions, lay counsellors from among people living with HIV (PLHIV), PE groups from local businesses, and provided health insurance awareness sessions when available (e.g. in South Africa and Kenya), and through family days, 1 December (commemorative day) events, and email sensitization/video forums.

With time, the family days were revised according to needs. Instead of a family awareness day for the whole family, they were separated into young adults’ day and parents’ day; both events included HIV testing for eligible persons. Awareness sessions always considered the informational needs of staff and were delivered in local languages. In some contexts, “domestic violence” or “homosexuality” came out as important topics to be addressed (e.g. in South Africa, Thailand, Kenya, Haiti), while they were absent in more religious or traditional settings (e.g. Sudan, Lao People’s Democratic Republic). A culture of openness and non-discrimination was promoted. Addressing possible stigma and discrimination was a daily issue [21].

### VCT and ART services

HFPs identified the best VCT and ART service providers (mainly local nongovernmental organizations or in rare cases private institutions), which were scarce in some remote settings. VCT services were outsourced to HIV prevention and treatment service providers:

- to guarantee privacy and confidentiality to the staff. The only information provided to the HFPs were anonymous data on employees and family members tested yearly and on the positives tests;
- to guarantee continuum of care and access to ART according to national protocols;
- to respect local legislation, standards, criteria and epidemiology;
- for sustainability as well as to strengthen the WPP system.

The particularities of the ICRC compared to other employers are the following:

- by its mandate, the ICRC is involved in fragile States and countries in conflict where health services are often endangered and there is reduced access to care, stocks or quality services but also by lack of update of national public health policies;
- the main ICRC activities are delivered far from capital cities, near frontlines, where there is often an extreme scarcity of health services;
- in permanent adaptation to the needs generated by the conflicts, the size of the staff fluctuates over the years;
- the ICRC always respects national health policies and norms and does not develop its own ART clinics. Employees are proposed VCT services in an existing clinic, validated by the ministry of health. This guarantees confidentiality, respect of national norms concerning CD4/viral load criteria for starting treatment, inclusion of staff in the national health system and statistics for continuum of care after possible layoffs;
- there is a common HR administrative system to all delegations of the ICRC, so similarities are found in all countries (e.g. salary classes, types of contract);
- as a humanitarian organization, the ICRC delivers high-quality services at the best prices, with no logic of competition with the other health service providers or transfer of foreign investment. Very few of the affected countries have private health insurance providers. Therefore, the ICRC insures its staff and family members itself, leading to a situation in which the employer is also the insurer.

The ICRC guarantees a continuation of health insurance coverage for one year after the end of contract for patients with chronic diseases. It is one of the responsibilities of the HFP to monitor the integration of former colleagues into functional national programmes over the years.

## Method

We carried out a retrospective analysis on data available over 10 years. VCT uptake is the main indicator that has been consistent throughout the years.

Some of the data came from HIV knowledge, attitude and practice (KAP) surveys established early in the relevant countries (e.g. in 2004 in Kenya, in 2005 in Ethiopia, in 2006 in Uganda, in 2008 Democratic Republic of the Congo and Burundi, and in 2010 in Zimbabwe) using a common questionnaire and showing similar results and limitations (Table 1).

**Table 1:** KAP results

• Good age representation of the ICRC staff, but lower completion rate in remote offices than in the capital city
• Good knowledge (mean score of 14.3 on a scale of 20) on HIV main modes of transmission except mother to child transmission where 40 % to 55 % of them did not know the way of preventing mother to child transmission. According to countries 3 (Kenya) to 16 % (Burundi) believed HIV is transmitted by witchcraft, and for 3 (Kenya) to 23 % (DRC) it is God’s punishment.
• Misconception as “birth control pills prevent HIV transmission, or mosquito transmission” were made by respectively 17.4 and 10.7 % (North Kenya) in some remote offices.
• Employees below 25 years had a significant difference in knowledge index. There is a confirmed correlation between level of education and knowledge index.
• According to countries, between 5 to 15 % of the staff reported having a diagnosed STI in the preceding 12 months and only 40 % agreed that having many sexual partners can lead to STI while 10 (Kenya) to 21 % (DRC) declared having more than two partners in the last 6 months (singles > married, but not related to number of days out of station)
• In average 70 % were willing to go for VCT, 65 % if done at the workplace with confidentiality.
• More than 80 % who were tested shared the results with someone, but disparities were documented in Kenya between the capital and remote office were, respective, 38 to 53 % were never tested due to fear.
• While 83 to 93 % declared themselves ready to take care of a PLHIV relative, 70 (North Kenya office) to 94 % (DRC) declared that a sick relative should remain a secret.
• Up to 90 % declared themselves comfortable discussing HIV/AIDS with a counsellor, only 70 % were ready to do it with their children and 65 % with their boss. Discussion about their own status was significantly lower (from 78 % with spouse to 12 % with the boss). 75 to 80 % agreed that PLHIV have an equal chance of recruitment or promotion at the ICRC
• Staff from remote offices were significantly considering themselves at low infection risk, while they were also scoring lower in knowledge index.
• In North Kenya, up to 14 % of the men and 20 % of women described having been forced to have sex.
• While few declared regular alcohol consumption, there was a correlation between daily alcohol consumption and reporting sex with a commercial sex worker.
• Condom perception and use showed better results for 25 to 39 years old. Persons younger than 25 years scored lower due to shame and misconception. The consistency of condom use was not assessed.
• More than 95 % saw a PLHIV dying of AIDS

A set of outcome indicators respecting national protocols was established (e.g. number of employees attending the awareness sessions, number of PE/staff), which are the same indicators as those used by the IFRC [22].

**Fig. 1:**
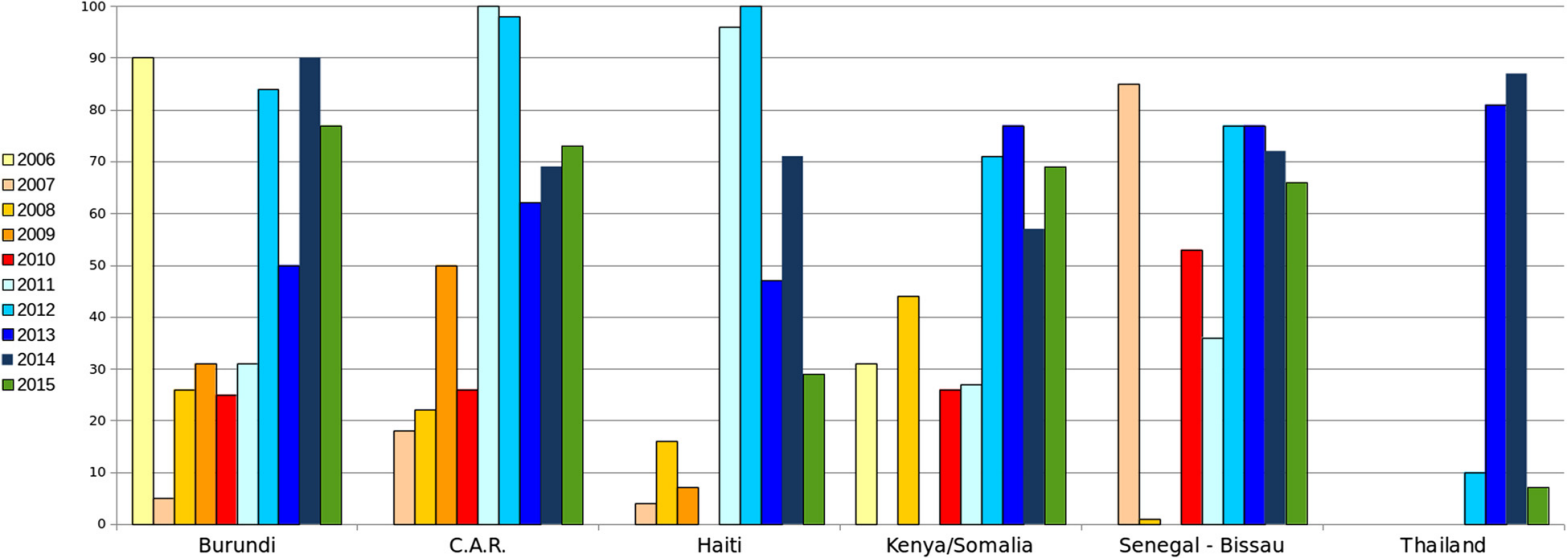
Annual VCT uptake in percentage of staff from 2006 to 2015; choice of countries

These KAP surveys eventually increased the acceptance of the HIV WPP but didn’t increase the VCT uptake. For example, the KAP survey in Kenya was carried out in 2004, the one in Uganda in 2006, and the one in Burundi in December 2008. Figure 1 illustrates the lack of correlation between the KAP surveys and the VCT uptake.

After initial awareness raising achieved through KAP, the approach moved to implementation of VCT at the workplace and supporting by all means possible the prevention of mother to child transmission (PMTCT).

Data collection concentrated on key performance indicators such as VCT uptake per delegation, PMTCT and financial data.

## Results

### Geographical expansion

The Table 2 illustrates the growing expansion of the programme, starting with two pilot projects in Kenya 2004 and Senegal in 2005. Finding local actors for KAP and VCT partnerships in respect of national policies was the main challenge of the first years. The numbers reported are percentages of staff knowing their HIV status on an annual basis. The HIV WPP started in 2004, so 2006 data are cumulative ones for 2004 to 2006. We put 0 for HIV WPP present without VCT. Mali and Niger were one single delegation originally, but became two separate delegations in 2014. The South Africa 2014 results were not communicated by the health insurance agency according to national WPP protocols. Some delegations (e.g. Angola) were closed as a result of conflict ending. Figure 3 illustrates the results from a selection of these countries.

**Table 2:**
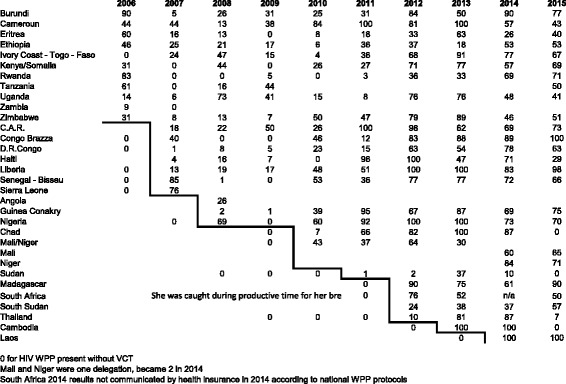
Expansion of the HIV WPP by its annual VCT uptake in percentage of staff

### Staff coverage and VCT uptake

Figure 1 illustrates different dynamics in representative delegations.

Burundi was an early successful programme thanks to the personality of the HFP, sharing his/her HIV positive status. Erosion is visible from 2007 to 2011, when the offering of testing and health coverage of other conditions boosted the VCT.

Continuum of care from VCT to access to treatment was offered in the Central African Republic, the HFP being a doctor working in both services. The leadership of the HIV-positive Head of Delegation boosted the VCT of 2011 and 2012.

The drop of VCT in Haiti is due to a security incident affecting the delegation in 2013, then a drastic reduction of staff in 2015. Awareness and mobilization were extremely country specific, with, for example, Haitians expressing themselves about HIV with a striking freedom.

The figures from the regional delegations of Kenya/Somalia and Senegal/Guinea Bissau illustrate the difficulties of keeping annual VCT mobilization. The family days were parties attended by numerous employees but very few VCT were taken during these events. The combination of VCT with other tests at the workplace during working hours is the reason for the visible increase in 2012.

The prevalence of HIV being low in Thailand, it was virtually impossible to interest the staff in knowing their status without a combination with other tests. While this approach was validated, the VCT uptake increased rapidly and was maintained in 2015.

**Fig. 2:**
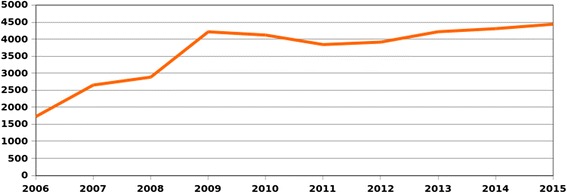
Number of ICRC staff covered by the HIV WPP since its beginning

Figure 2: The increase is due to growing number of countries involved and regular increase of staff.

**Fig. 3:**
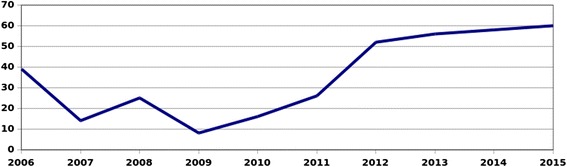
Annual VCT uptake in percentage of ICRC staff since the beginning of the HIV WPP

Figure 3: 2006 VCT values are persons tested since the beginning of the programme (2004 to 2006) while 2007–2015 values are a calculation of annual VCT uptake. In order to avoid discrimination, we proposed that persons living with HIV should queue with their colleagues and receive, in the privacy of the medical consultation, a free voucher for CD4/viral load (VL) testing. The lowest annual VCT uptake was 9 % while 60 % was reached in 2015.

### Prevention of mother to child transmission

**Fig. 4:**
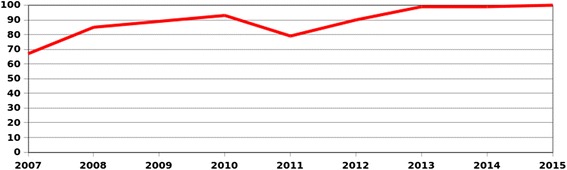
Percentage of pregnant women knowing their HIV status on annual basis since the beginning of the HIV WPP

Figure 4 shows that access to PMTCT for staff and spouses rose from 67 % in 2007 to 100 % in 2015.

HFP reinforced the anchorage of the programme in national health policies, which were developing at their own pace.

### Financial split

**Fig. 5:**
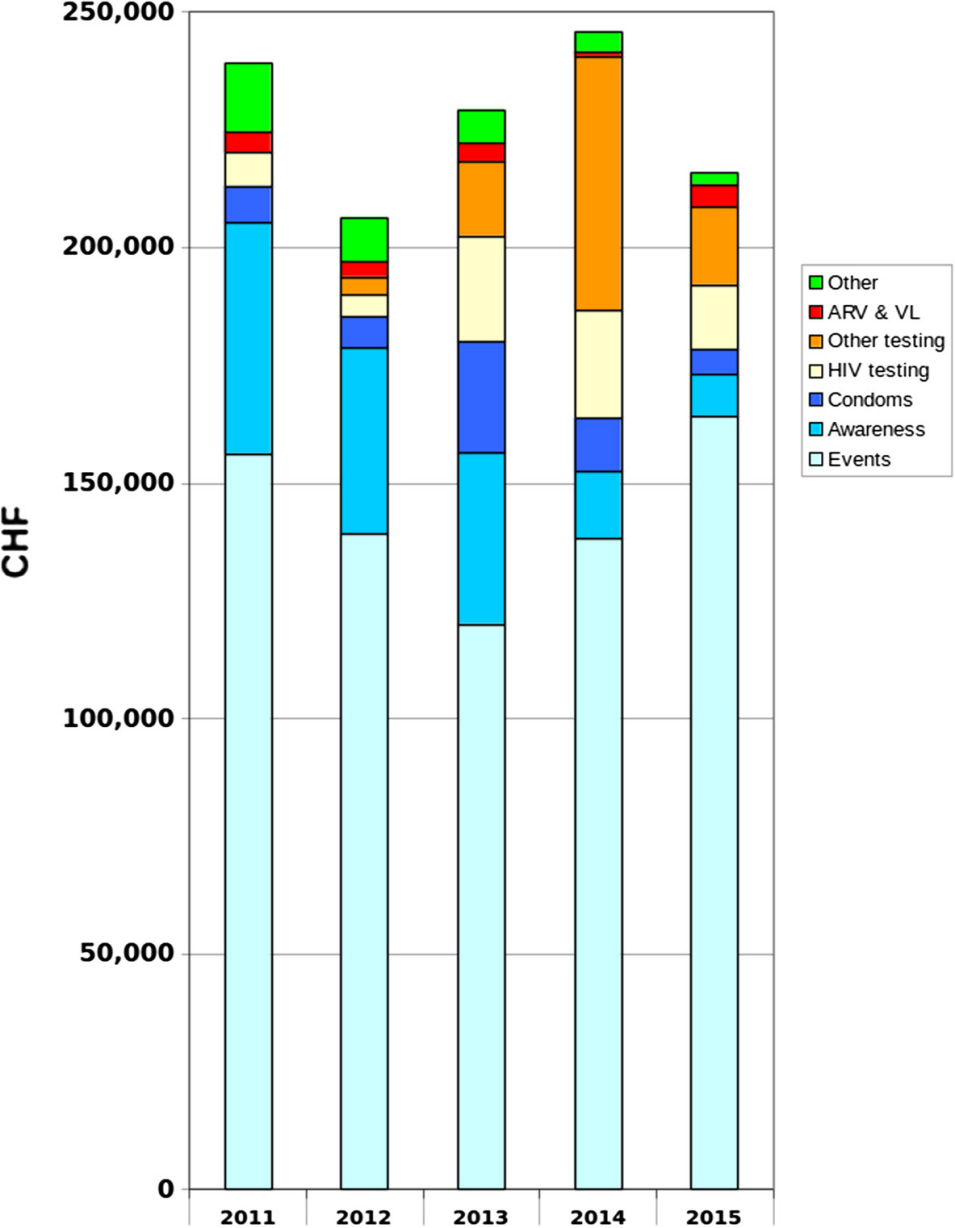
HIV WPP financial split

Figure 5 shows that since 2011, we aimed to decrease the cost of awareness (social events, material) in favour of testing (HIV testing, VL, CD4, hepatitis B vaccination, other screening tests) and ART.

## Discussion

The HIV WPP had to overcome unique challenges, similar to those facing multinational companies [23], due to the geographical disparity of the settings in which the programme took place. For example, in 2014, 767 employees in the Democratic Republic of the Congo were spread across eight locations, while in South Africa the 24 employees all worked in the capital, Pretoria.

Another challenge is related to working in conflict zones. In the last three years, we anticipated and prevented national or regional stock outs of ART drugs (e.g. in Burundi), HIV tests or access to annual CD4 count (e.g. in the Democratic Republic of the Congo), or destruction and looting of ART centres (e.g. in the Central African Republic). This increased drastically the trust placed in the programme by staff.

### External mainstreaming

An indirect effect of the WPP was the external mainstreaming conducted by sensitized and trained workers in their communities with a positive effect on communities in terms of knowledge and attitude. In Senegal, for example, proactive outreach in the community was undertaken in collaboration with the Senegalese Red Cross to prevent HIV/AIDS in migrant women. A positive effect on the communities was also seen in Sudan, where the ICRC’s HIV WPP was the first WPP in the country. This contributed to the fight against stigma and discrimination, in disseminating information and inciting people to test for HIV. It was also the first programme to make condoms available, while respecting national law, contributing to the reduction of HIV transmission. Outreach of the programme to families with adolescents and making treatment available, which drastically reduce the spread of the virus when ART is correctly taken, played a positive role in HIV prevention.

### The success of de-stigmatization of HIV testing over the last years

Due to a low initial VCT uptake, mainly due to the belief that people taking an HIV test put themselves at risk or are promiscuous, we introduced in 2010 three different strategies as incentives and rewards for testing, after reviewing various strategies in the literature [24, 25]:

- Communication campaigns for developing people’s ownership of their own health: “Be responsible for you and your family” was the motto of all awareness-raising activities (posters, talks, online articles). This implies that you do not take an HIV test on suspicion any longer, but do so as a responsible person or parent so that if you are negative you can work at remaining negative and if you are positive, early treatment will be offered to you at no cost.
- Annual health status screening packages including VCT. We reviewed the literature in order to select meaningful and contextualized screening tests (blood pressure, body mass index, cervical smear, breast examination, fasting blood sugar and hepatitis B). We only did these tests in contexts where follow-up and care would be provided in case of positive results.
- The three regional coordinators, on request, accompanied colleagues living with HIV to the ART centres and during hospitalization. Covering ART shortages for ICRC staff and their families increased WPP acceptance and continuum of care because confidentiality was always maintained.

### Evolution of monitoring and evaluation (M&E)

While reviewing eight major studies based in the workplace, the Cochrane Collaboration [26] illustrated the numerous risks of bias and the difficulties in comparing indicators, despite previous literature urging that M&E operational challenges be addressed [27, 28].

We regularly assessed the quality of testing, tools, methodology and confidentiality in each delegation. We introduced key performance indicators, in respect of ILO standards, to document mortality, morbidity, infections related to HIV, medical costs and absenteeism. Nonetheless, the diversity of ICRC settings, the respect of patient confidentiality and the proven long-term benefit of VCT and ART in the literature [17, 18, 29–32] and the documented effectiveness of WPP [15, 23, 33–36] made these indicators of reduced interest with time. It was then proven by others that it is more efficient to implement an HIV WPP than to lose and to replace people.

Due to confidentiality concerns and the policy of referring people to existing external ART services, ART uptake and retention and treatment adherence could not be documented consistently in the 35 contexts. In exceptional cases, at the request of the family, the HFP was called in to persuade PLHIV to start or continue with treatment.

Since 2009, we decided to provide annual VCT at the workplace and encountered initial resistance despite its recognized benefits [37]. Afraid of social turmoil, or convinced that the national programmes were “so well in place in the country”, some line managers, who had to address competing operational priorities in conflict zones, had to be convinced before accepting the implementation of this strategy in their place of work. This internal resistance, also documented in other organizations [38], contrasted with the involvement of the line manager in the Heineken brewery in Bukavu, who led by example in HIV testing, knowledge of condom use etc., with condoms being available on his own desk.

Regular visits to the main HIV stakeholders in the different countries (ministries of health, Global Fund, UNAIDS representatives, PLHIV groups and the main VCT-ART providers) allowed the sharing of experiences, adaptation of the programme, and benchmarking.

External evaluations were done in 2005 and 2010, resulting in regular proposed improvements. Benchmark visits generated changes in 2011 and 2012.

The ICRC HIV workplace policy was compared to ISO 26000 standards on corporate social responsibility [13] by four masters students from Zurich University in early 2014 [Haering A, Mayer I, Sager R, Sayer S. A Framework for HIV Workplace Programs based on Social responsibility Issues. Switzerland: University of Zurich; 2014. Unpublished master manuscript], which compared the ICRC WPP with the ILO standards in the world of work [9, 39], finding no substantial missing points.

## Conclusions

The ICRC HIV WPP shows the feasibility of running such a programme in an operational institution working in conflict zones. This success has been possible through leadership and commitments at different levels, from the ICRC Assembly to the staff benefiting from the programme, and has been maintained despite the relatively high turnover of staff within the ICRC. Positive results of external mainstreaming of the WPP are strong arguments to develop more WPP and contribute to the UNAIDS Fast-Track 90-90-90 objectives.

After years of awareness rising, we have been able to implement VCT uptake in the workplace, and to expand geographically (to 25 countries in 2013) and thematically to promote health on a larger scale.

Indeed, the HIV WPP has to continuously adapt to evolving contexts, and keep in mind the safety of staff (Primum Non Nocere). The 2015 VCT uptake results (60 %) might be difficult to reach in the future due to political turmoil in many countries. In 2014, the Ebola crisis in Liberia and in Guinea jeopardized the blood sampling of VCTs. New interreligious (e.g. Central African Republic) and/or interethnic (e.g. South Sudan) conflicts have created new boundaries within countries and restricted access of ICRC staff to HIV care.

As part of the ICRC’s social responsibility commitments, we aim to replicate the HIV WPP structure in other ICRC operational contexts (e.g. in the Syrian Arab Republic and Iraq) within the framework of the ICRC’s duty of care, which in some cases in such contexts focuses other health priorities, like chronic conditions and mental health, rather than on HIV.

## Abbreviations

AIDS: Acquired Immune Deficiency Syndrome
ART: Antiretroviral therapy
CAR: Central African Republic
CD4: Lymphocytes type CD4
GIZ: Deutsche Gesellschaft für Internationale Zusammenarbeit: German society for International Cooperation
HFP: Health Focal Point
HIV: Human Immunodeficiency Virus
HR: Human Resources
ICRC: International Committee of the Red Cross
IFRC: International Federation of Red Cross and Red Crescent Societies
ILO: International Labour Organization
KAP: Knowledge, Attitude and Practices
M&E: Monitoring & Evaluation
PE: Peer Educators
PLHIV: People Living with HIV
PMTCT: Prevention Mother to Child Transmission
STI: Sexually Transmitted Infection
UNAIDS: United Nations for AIDS
VCT: Voluntary Counselling (Confidential) Testing
VL: Viral Load
WPP: Workplace Programme
